# The ethics of staying: Medicine at the limits of intervention

**DOI:** 10.1017/S1478951526102387

**Published:** 2026-04-20

**Authors:** João Carlos Geber-Júnior

**Affiliations:** Hospital das Clínicas da Faculdade de Medicina da Universidade de São Paulo, São Paulo, SP, Brazil

Medicine is often defined by what it can do. Yet some of the most consequential moments in clinical care arise precisely when there is little left to do. At those limits – when treatment options narrow and certainty fades – the ethical character of medicine becomes visible in a different form.

Modern medicine is built upon an extraordinary capacity to intervene. Diagnostic technologies reveal disease with unprecedented precision, therapeutic innovations extend survival in conditions once considered fatal, and clinical training reinforces the imperative to act. Within this culture of intervention, physicians are taught to understand their role primarily through technical response: identify pathology, apply treatment, alter the course of disease.

Yet the lived experience of serious illness reveals a more complex landscape. Many of the situations that most profoundly shape the experience of patients and families unfold precisely where medicine’s ability to intervene becomes uncertain or limited. Prognostic ambiguity, progressive disease, existential distress, and relational suffering are not peripheral phenomena in clinical practice – they are central features of it.

These circumstances raise a deeper ethical question: what does it mean to care when intervention is no longer the primary therapeutic tool?

Palliative care has long engaged with this question by emphasizing that the goals of medicine extend beyond cure to include the relief of suffering and the preservation of dignity. From this perspective, the limits of intervention do not signal the end of care but rather a transformation in its meaning.

## Suffering and the integrity of the person

One of the most influential contributions to this understanding came from Eric Cassell, who argued that suffering arises when illness threatens the integrity of the person rather than the body alone (Cassell 1982). Disease may damage tissues, but suffering emerges when illness disrupts identity, relationships, meaning, and the imagined future.

Medical anthropology further expanded this perspective. Arthur Kleinman emphasized that illness is experienced within narratives that shape how suffering is interpreted and communicated (Kleinman 1988). At the same time, Cicely Saunders introduced the concept of *total pain*, recognizing that physical, psychological, social, and spiritual sufferings are inseparable dimensions of serious illness (Saunders 2001).

Within these frameworks, the clinical encounter becomes more than a site of diagnosis and treatment. It becomes a relational space in which suffering is recognized, interpreted, and – when possible – shared.

## The culture of intervention

Despite this broader understanding of suffering, contemporary medical culture continues to privilege action. Clinical competence is frequently associated with decisiveness, procedural skill, and the capacity to modify biological processes.

This orientation has produced extraordinary therapeutic advances. Yet it also generates an implicit expectation that meaningful care must always take the form of intervention.

When suffering persists despite treatment, clinicians may experience uncertainty or discomfort. The absence of effective intervention can feel like a professional void. In such contexts, clinical encounters risk narrowing to what remains measurable – laboratory values, imaging findings, or pharmacologic adjustments.

Patients often experience these moments differently. Their central concern is rarely whether every possible intervention has been attempted. Instead, it is whether someone remains present as illness unfolds.

## The ethics of staying

To remain present with patients during uncertainty or decline may appear deceptively simple. Yet within contemporary medical culture it represents a profound ethical commitment.

Staying requires clinicians to resist the impulse to withdraw when illness cannot be easily altered. It involves acknowledging uncertainty, tolerating emotional complexity, and maintaining relational engagement even when technical solutions are limited.

Patients frequently recognize this distinction. Expressions of gratitude often arise not from technical explanations alone but from the perception that the clinician did not disappear when circumstances became difficult.

Previous reflections from clinical practice suggest that palliative care may be understood less as a referral triggered by prognosis and more as a clinical stance grounded in time, relational presence, and the honest acknowledgment of medicine’s limits (Geber-Junior and Forte 2025a). Similarly, communication may fail when it becomes a scripted intervention rather than a relational encounter capable of holding uncertainty and personhood (Geber-Junior and Forte 2025b).

Other reflections suggest that suffering itself may become a teacher, revealing dimensions of illness that cannot be captured through diagnostic categories alone (Geber-Junior 2025a). Encounters with patients facing progressive disease further demonstrate that the most meaningful form of care may lie not in additional intervention but in the willingness to remain present through uncertainty (Geber-Junior 2025b).

More recently, reflection on sadness in serious illness has highlighted how emotional responses to threatened meaning may be prematurely medicalized within clinical environments, further reinforcing the importance of relational presence in palliative care (Milan-Youssef and Geber-Junior 2026).

Taken together, these observations suggest that presence itself may function as a form of care.

## Presence and relational care

The importance of relational presence has been widely explored within palliative care scholarship. Research on dignity in serious illness has shown that patients value clinicians who acknowledge their personhood beyond disease, recognizing identity, meaning, and relational ties.

Work by Chochinov demonstrated that threats to dignity frequently arise when illness undermines a patient’s sense of identity (Chochinov 2002). Within such contexts, clinician presence and acknowledgment of personhood can significantly influence the experience of care.

Similarly, meaning-centered approaches developed by Breitbart and colleagues emphasize that existential concerns – including meaning, purpose, and legacy – are central dimensions of suffering in serious illness (Breitbart et al. 2015).

These perspectives reinforce an essential insight: suffering is experienced by persons, not by bodies. Therefore, addressing suffering requires more than technical intervention; it requires relational engagement.

## Medicine at its limits

The limits of medicine are often described biologically – moments when disease progresses despite treatment. Yet there is another limit that concerns the scope of clinical responsibility.

If medicine defines care exclusively through intervention, its ethical reach inevitably contracts when intervention becomes impossible. The ethics of staying challenges this assumption.

It suggests that medicine retains responsibility even when its technical power is constrained. In these moments, the clinician’s task shifts from altering the trajectory of disease to protecting the integrity of the person who bears it.

Presence thus becomes a form of professional competence: the capacity to remain engaged, attentive, and relationally available even when clinical certainty is absent.

## Conclusion

Modern medicine’s extraordinary capacity to intervene has transformed the treatment of disease. Yet the experience of serious illness continues to reveal situations in which intervention alone cannot address the full scope of suffering.

In these moments, the ethical foundations of medical care become visible.

To stay with patients during uncertainty, decline, or existential distress is not a passive gesture. It is an act of responsibility that affirms the dignity of persons when illness threatens to reduce them to diagnoses alone.

If the power of medicine is measured by its ability to intervene, its moral depth may be revealed by its willingness to remain present when intervention reaches its limits.

In this sense, the ethics of staying represents not the absence of care but one of its most essential forms.
